# The effect of normobaric hypoxia on acute exercise-induced changes in blood sphingoid base-1-phosphates metabolism in cyclists

**DOI:** 10.5114/biolsport.2024.131414

**Published:** 2023-09-21

**Authors:** Katarzyna Hodun, Miłosz Czuba, Kamila Płoszczyca, Jerzy Sadowski, Józef Langfort, Adrian Chabowski, Marcin Baranowski

**Affiliations:** 1 Department of Physiology, Medical University of Białystok, Białystok, Poland; 2 Faculty of Rehabilitation, Józef Piłsudski University of Physical Education in Warsaw, Warsaw, Poland; 3 Department of Kinesiology, Institute of Sport – National Research Institute, Warsaw, Poland; 4 Faculty of Physical Education and Health, Józef Piłsudski University of Physical Education in Warsaw, Warsaw, Poland; 5 Department of Sports Theory, Jerzy Kukuczka Academy of Physical Education, Katowice, Poland

**Keywords:** Ceramide, Red blood cells, Simulated altitude, Sphinganine-1-phosphate, Sphingomyelin, Thrombocytes

## Abstract

Extracellular sphingosine-1-phosphate (S1P) emerged as an important regulator of muscle function. We previously found that plasma S1P concentration is elevated in response to acute exercise and training. Interestingly, hypoxia, which is commonly utilized in training programs, induces a similar effect. Therefore, the aim of the current study was to determine the effect of normobaric hypoxia on exercise-induced changes in blood sphingolipid metabolism. Fifteen male competitive cyclists performed a graded cycling exercise until exhaustion (GE) and a simulated 30 km individual time trial (TT) in either normoxic or hypoxic (FiO_2_ = 16.5%) conditions. Blood samples were taken before the exercise, following its cessation, and after 30 min of recovery. We found that TT increased dihydrosphingosine-1-phosphate (dhS1P) concentration in plasma (both HDL- and albumin-bound) and blood cells, as well as the rate of dhS1P release from erythrocytes, regardless of oxygen availability. Plasma concentration of S1P was, however, reduced during the recovery phase, and this trend was augmented by hypoxia. On the other hand, GE in normoxia induced a selective increase in HDL-bound S1P. This effect disappeared when the exercise was performed in hypoxia, and it was associated with reduced S1P level in platelets and erythrocytes. We conclude that submaximal exercise elevates total plasma dhS1P concentration via increased availability of dihydrosphingosine resulting in enhanced dhS1P synthesis and release by blood cells. Maximal exercise, on the other hand, induces a selective increase in HDL-bound S1P, which is a consequence of mechanisms not related to blood cells. We also conclude that hypoxia reduces post-exercise plasma S1P concentration.

## INTRODUCTION

Sphingoid base-1-phosphates represent a subgroup of sphingolipids known for their high biological activity. They are produced via phosphorylation of free sphingoid bases by the enzyme sphingosine kinase (SphK). The two best known members of this subgroup are sphingosine-1-phosphate (S1P) and dihydrosphingosine-1-phosphate (dhS1P), which are synthesized from sphingosine and dihydrosphingosine, respectively. Sphingosine is a product of ceramide degradation by ceramidase, whereas dihydrosphingosine is an intermediate in the de novo sphingolipid synthesis pathway. S1P and dhS1P are irreversibly degraded by S1P lyase, or dephosphorylated back to free sphingoid bases by S1P phosphatase or nonspecific lipid phosphate phosphohydrolase [1].

Sphingoid base-1-phosphates can be exported to the extracellular space by facilitator superfamily transporter 2b, spinster homolog 2, and several members of the ATP-binding cassette transporter family [2–4]. It is generally accepted that most of their biological effects are induced extracellularly via binding to a family of five specific membrane G-protein coupled receptors (S1PRs) [5].

Sphingoid base-1-phosphates are found in relatively high concentrations in the human plasma, where they are transported mainly by high-density lipoprotein (HDL) and albumin. In addition, large quantities of S1P and dhS1P are stored in red blood cells (RBCs) and platelets, which are characterized by high SphK activity and low expression of enzymes responsible for their degradation. RBCs release sphingoid base-1-phosphates constitutively, and were found to be the major source of circulating S1P and dhS1P. Platelets, on the other hand, secrete them only upon activation. Significant amounts of sphingoid base-1-phosphates are also constitutively released to the circulation by vascular endothelial cells [6].

There is an increasing body of evidence indicating that extracellular S1P is an important regulator of muscle function, likely involved in its adaptation to exercise. It affects the excitation-contraction coupling in a way resulting in an anti-fatigue effect, stimulates muscle regeneration via activation of satellite cells, enhances mitochondrial respiration, and induces angiogenesis [7].

We have previously found that concentration of sphingoid base-1-phosphates in the plasma is elevated in response to both acute exercise and training [8, 9]. Interestingly, hypoxia, which is commonly utilized in athletes’ training programs due to its performance benefits, was recently shown to induce a similar effect on circulating S1P and dhS1P at rest [10]. Stimulation of A2B adenosine receptor in RBCs was found to induce activation of SphK and accumulation of S1P, indicating that adenosine may mediate the effect of hypoxia on circulating sphingoid base-1-phosphates metabolism [11]. Accumulation of S1P and dhS1P in response to hypoxia was also observed in human cerebral endothelial cell line, which was a consequence of reduced S1P lyase activity [12].

Altitude training has become a standard training protocol in many sports disciplines, including cycling, to increase exercise capacity at sea level, to acclimatize prior to competitions at altitude, or before ascending to altitude [13]. A sudden exposure to the hypoxic environment, or a longer stay at elevated altitude, induces numerous adaptations which can lead to improved athletes’ performance at sea level. These mechanisms are generally attributed to either hematological, cardiovascular, or ventilatory effects of altitude training [14–16]. In addition, acute and chronic exposure to hypoxia induces serval metabolic consequences in the body and combined with exercise presents an enormous challenge for athletes [17]. The process of adaptation to hypoxia is important not only for professional athletes. Due to the growing popularity and availability of high mountain tourism and mountain-related forms of recreation, issues related to the adaptation of the human body to hypoxia are becoming important to a growing number of people.

Therefore, considering the above facts, the aim of the current study was to determine the effect of normobaric hypoxia on acute changes in blood sphingolipid metabolism induced by exercise. Our hypothesis was that the increase in plasma sphingoid base-1-phosphates concentration in response to acute exercise is augmented by a concomitant exposure to hypoxia, which could mediate the beneficial effects of hypoxic training. In the present study we used the exercise protocols, namely graded exercise until exhaustion and 60 min of submaximal exercise, which we previously found to induce acute changes in the metabolism of circulating sphingoid base-1-phosphates [8, 18].

## MATERIALS AND METHODS

### Experimental design

The investigation conforms to the ethical norms and standards in the Declaration of Helsinki and was approved by the Bioethics Committee at the Medical University of Bialystok (approval no. R-I-002/325/2019). Each subject signed an informed consent before inclusion in the study.

Fifteen highly-trained male competitive cyclists (age 25.4 ± 8.4 years, BMI 21.6 ± 1.8 kg/m^2^, body fat content 9.2 ± 2.1%, V_O2max_ 61.4 ± 3.1 mL/kg/min) with an average training experience of 6.3 ± 2 years participated in the study. All subjects possessed a valid medical certificate confirming the absence of contraindications to the practice of competitive sport activity.

The athletes were instructed to maintain their regular diet and supplementation throughout the experiment, and were asked to abstain from caffeine intake during 24 h preceding each test. All participants arrived at the facility one day before the start of each test series and consumed the same meals throughout their stay (40 kcal/ kg/d, 50% carbohydrates, 20% proteins, and 30% fats).

The subjects were tested during two sessions, separated by two weeks, in either normoxic or hypoxic (FiO_2_ = 16.5%, equivalent to 2,000 m asl) conditions applied in a random order. The athletes were blinded to exercise conditions. The tests were performed in a room equipped with a normobaric hypoxia system (AirZone 25, Air Sport, Międzyzdroje, Poland) allowing to manipulate oxygen concentration in the air. Temperature (19°C), humidity (50%), and CO2 concentration (700–800 ppm) were controlled and held constant.

In the morning of the first day of each session, two hours after a light breakfast, the subjects performed graded cycling exercise until volitional exhaustion (GE). Volitional exhaustion was defined as the participants’ inability to continue exercising, despite strong encouragement by the testing staff. The exercise started with a work-load of 120 W, which was then increased by 40 W every 3 min. The total duration of exposure to hypoxia during this test was ~35 min.

On the next day, following 24 h of rest (two hours after a light breakfast), the athletes performed a simulated 30 km individual time trial (TT) in a mountainous terrain. The TT was preceded by a 15 min warm-up, carried out according to the athletes’ individual preferences, under oxygen concentration corresponding to the main exercise. The total duration of exposure to hypoxia during this test was ~90 min. Both tests were performed on subjects’ personal bicycles connected to an electromagnetic bicycle trainer (Cyclus 2, RBM Elektronik-Automation GmbH, Leipzig, Germany). The athletes were allowed to consume water ad libitum during each exercise. The oxygen saturation of arterial blood (SpO_2_) and heart rate were measured using the WristOx2 pulse oximeter (Nonin Medical Inc., Plymouth, MN).

### Blood fractionation

During each test day blood samples were taken from the antecubital vein at three time points: before the onset of exercise, within 5 min following its cessation, and after 30 min of recovery in normoxia. Blood was put on ice immediately after sampling into 4 mL EDTA tubes. Erythrocytes, platelets, and platelet-free plasma were isolated by sequential centrifugation. Temperature was maintained at 4°C throughout whole procedure to prevent sphingoid base-1-phosphates release from erythrocytes [19]. Separated blood cells were resuspended in cold PBS and flash frozen in liquid nitrogen.

HDL were isolated from the platelet-free plasma by sequential flotation ultracentrifugation in NaBr solution in a Sorvall RC M120 GX ultracentrifuge (Thermo Fisher Scientific, Waltham, MA) equipped with a S120-AT2 rotor as described by Havel et al. [20] with some modifications. Briefly, plasma was centrifuged at 120.000 rpm for 85 min at 8°C at a density of 1.006 g/mL to remove very low-density lipoprotein, for 155 min at a density of 1.063 g/mL to remove low-density lipoprotein, and for 260 min at a density of 1.21 g/mL to obtain HDL. After the last centrifugation step lipoprotein-depleted plasma containing albumin was recovered from the bottom of the tube. To avoid oxidation of lipoproteins, 0.3 mM EDTA was present in all preparation steps. Densities of NaBr solutions used for density adjustments were checked with a digital densitometer (Densito 30P, Mettler Toledo, Columbus, OH). All samples were stored at (–80°C) until analysis.

### Sphingoid base-1-phosphate release from erythrocytes

Cold erythrocyte concentrate was resuspended in precooled plasma isolated from the same blood sample to obtain hematocrit of ~45%. To determine the baseline sphingoid base-1-phosphate concentration in the medium, an aliquot of the erythrocyte suspension was immediately transferred to a fresh tube and centrifuged at 4°C. The supernatant was then flash frozen in liquid nitrogen. The remaining portion of the erythrocyte suspension was incubated for 20 min at 37°C in 5% CO_2_. Samples were then immediately cooled on ice and the medium was separated by centrifugation at 4°C. The rate of S1P and dhS1P release was calculated by subtracting their basal concentration in the medium from the value measured after the incubation.

### Sphingolipid analysis

The content of S1P, dhS1P, sphingosine, dihydrosphingosine, ceramide, and dihydroceramide was determined as previously described in detail [21]. Briefly, lipids were extracted from samples in the presence of internal standards (C17-sphingosine and C17-S1P, Avanti Polar Lipids, Alabaster, AL). An aliquot of the lipid extract was transferred to a fresh tube with pre-added N-palmitoyl-D-erythro-sphingosine (C17 base, Avanti Polar Lipids) as an internal standard, and then subjected to alkaline hydrolysis to deacylate ceramide and dihydroceramide to sphingosine and dihydrosphingosine, respectively. The amounts of S1P and dhS1P were determined indirectly after dephosphorylation to sphingosine and dihydrosphingosine, respectively, with the use of alkaline phosphatase. Free sphingosine and dihydrosphingosine, dephosphorylated sphingoid base-1-phosphates, and sphingoid bases released from ceramide and dihydroceramide were then converted to their o-phthalaldehyde derivatives and analyzed using a UPLC system (Nexera, Shimadzu Corp., Kioto, Japan) equipped with a fluorescence detector (RF-20Axs) and a C18 reversed-phase column (Reproshell ODS-1, 2.7 μm, 125 × 3 mm, Dr Maisch, Ammerbuch, Germany). The isocratic eluent composition of acetonitrile:water (84:16, v/v) and a flow rate of 0.4 mL/min were used. Column temperature was maintained at 30°C.

For sphingomyelin analysis, lipids were extracted from erythrocytes and platelets by the method of Folch. Next, sphingomyelin was separated by thin-layer chromatography using the method described by Mahadevappa et al. [22]. The gel bands corresponding to the sphingomyelin standard were scrapped off the plates and transferred into fresh tubes containing pentadecanoic acid as an internal standard. Sphingomyelin fatty acids were then transmethylated in the presence of 14% boron trifluoride in methanol at 100°C for 90 min. The resulting methyl esters of palmitic, stearic, arachidic, behenic and lignoceric acid were analyzed by means of gas-liquid chromatography using a Hewlett-Packard 5890 Series II system equipped with a double flame ionization detector and Agilent (Santa Clara, CA) CP-Sil 88 capillary column (100 m, 0.25 mm i.d.). The content of sphingomyelin is presented as the sum of all analyzed individual fatty acid methyl esters.

Hemoglobin concentration was determined using Drabkin’s reagent kit (Sigma, Schnelldorf, Germany). Protein concentration was measured with the BCA protein assay kit (Sigma). Bovine serum albumin (fatty acid free, Sigma) was used as a standard.

### Statistical analysis

All data are presented as means ± SD. Statistical comparisons were made by using two-way ANOVA (with one factor being oxygen concentration during exercise, and the other factor being time) followed by Student’s t-test for paired samples. Correction for multiple comparisons was performed using Benjamini-Hochberg procedure. P < 0.05 was considered statistically significant.

## RESULTS

### General exercise and subjects characteristics

The average duration of the GE in normoxia and hypoxia was 20.2 ± 1.9 and 18.0 ± 1.9 min, respectively. The maximal work rate during the test performed under normoxic conditions was 5.1 ± 0.3 W/kg of body weight, whereas in hypoxia it amounted to 4.6 ± 0.4 W/kg. The heart rate at the point of volitional exhaustion was 193 ± 8 and 190 ± 9 bpm in normoxia and hypoxia, respectively. The resting SpO_2_ under normoxia was 98.0 ± 0.8, and during the final stage of the GE it was reduced to 91.9 ± 3.0%. In hypoxia, the SpO_2_ amounted to 93.3 ± 3.4 at rest, and 84.3 ± 5.4% at the point of exhaustion.

The duration of the TT in normoxia was 61.1 ± 5.4 min, whereas in hypoxia it increased to 63.7 ± 3.0 min. The average work rate during this test amounted to 3.6 ± 0.3 and 3.3 ± 0.2 W/kg of body weight under normoxic and hypoxic conditions, respectively. The heart rate was 176 ± 9, 177 ± 8, and 179 ± 10 bpm at 10, 20, and 30 km of the TT in normoxia, respectively. During the test performed under hypoxic conditions the corresponding heart rates were 174 ± 11, 176 ± 13, and 180 ± 12 bpm. In the normoxic TT, the SpO_2_ values at rest, and at 10, 20, and 30 km of the test were 97.8 ± 2.0, 94.5 ± 2.4, 94.1 ± 1.7 and 93.6 ± 2.3%, respectively. Under hypoxic conditions the corresponding SpO_2_ values were 93.3 ± 3.8, 85.6 ± 3.8, 86 ± 4.2 and 86.5 ± 2.7%.

### Graded exercise until volitional exhaustion

**FIG. 1:**
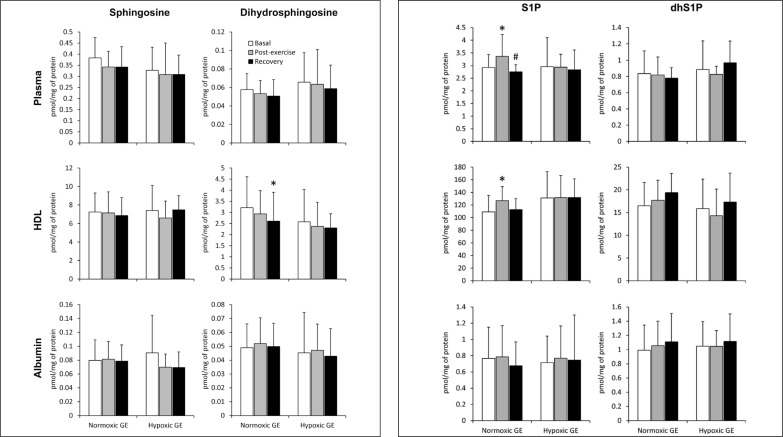
The content of sphingolipids in the plasma, as well as in its high-density lipoprotein (HDL) and albumin fractions sampled before, immediately after, and following 30 min of recovery from a graded cycling exercise until volitional exhaustion (GE) performed either in normoxia or at a simulated altitude of 2,000m asl (n=15). The results are means ± SD. Note: * – p<0.05 vs. the basal value, # – p<0.05 vs. the post-exercise value. HDL – high-density lipoprotein, dhS1P – dihydrosphingosine-1-phosphate, S1P – sphingosine-1-phosphate.

The total plasma S1P concentration increased in response to GE performed under normoxic conditions, which resulted entirely from the rise in the content of the HDL-bound pool. This effect was, however, transient and disappeared following 30 min of rest (Fig. 1). In addition, compared to the basal values, the content of the HDL-bound dihydrosphingosine was reduced (Fig. 1), whereas that of dhS1P in platelets (Table 1) was increased after 30 min of recovery from the exercise. In erythrocytes statistically significant changes induced by normoxic GE were observed only for ceramide and dihydroceramide levels, which were reduced below the basal values following 30 min of recovery (Table 1). The rate of sphingoid base-1-phosphates release from RBCs was not affected by the exercise (Fig. 3).

**TABLE 1:** The content of sphingolipids in erythrocytes and platelets sampled before, immediately after, and following 30 min of recovery from a graded cycling exercise until volitional exhaustion (GE) performed either in normoxia or at a simulated altitude of 2,000 m asl (n = 15).

	Normoxic GE			Hypoxic GE		

	Basal	Post-ex.	Recovery	Basal	Post-ex.	Recovery
**Erythrocytes (pmol/mg Hb)**						
Sphingosine	0.29 ± 0.1	0.294 ± 0.123	0.274 ± 0.1	0.307 ± 0.066	0.299 ± 0.061	0.326 ± 0.062
dhSph	0.024 ± 0.008	0.034 ± 0.017	0.033 ± 0.018	0.034 ± 0.012	0.035 ± 0.01	0.039 ± 0.011
S1P	4.29 ± 1.05	4.51 ± 1.23	4.08 ± 1.11	5.38 ± 1.15	4.57 ± 0.97^*^	5.04 ± 1.27^#^
dhS1P	1.92 ± 0.66	2.25 ± 0.94	2.23 ± 0.76	2.71 ± 0.95	2.2 ± 0.46	2.85 ± 1.17
Ceramide	35.1 ± 8.5	37.6 ± 10.3	28.1 ± 8.2^*^^#^	41.8 ± 10.6	42.9 ± 9.5	43.7 ± 10.7
dhCer	2.02 ± 0.97	2.06 ± 0.82	1.57 ± 0.67^*^^#^	2.34 ± 0.71	2.53 ± 1.03	2.63 ± 1.14
Sphingomyelin	1260 ± 140	1224 ± 133	1202 ± 121	1390 ± 172	1295 ± 128^*^	1429 ± 224^#^

**Platelets (pmol/mg protein)**						
Sphingosine	51.5 ± 34	51.7 ± 42.2	60.2 ± 50	60.5 ± 29	51.9 ± 40.2	51.9 ± 37.2
dhSph	11.5 ± 6.5	11.9 ± 9.2	13.6 ± 10.5	13.7 ± 6.6	12.8 ± 9.8	13.6 ± 8.7
S1P	536 ± 209	493 ± 217	560 ± 223	720 ± 181	565 ± 235^*^	564 ± 212^*^
dhS	1P 214 ± 96	211 ± 100	254 ± 106^#^	309 ± 69	272 ± 128	277 ± 112
Ceramide	944 ± 538	784 ± 241	771 ± 256	967 ± 220	890 ± 404	973 ± 381
dhCer	516 ± 274	429 ± 174	444 ± 198	576 ± 165	507 ± 204	543 ± 199
Sphingomyelin	19912 ± 4912	17553 ± 4329	18655 ± 3843	22607 ± 3755	19782 ± 5444	22564 ± 4900

The results are means ± SD.

* – p < 0.05 vs. the basal value,

# – p < 0.05 vs. the post-exercise value.

S1P – sphingosine-1-phosphate, dhCer – dihydroceramide, dhS1P – dihydrosphingosine-1-phosphate, dhSph – dihydrosphingosine, Hb – hemoglobin.

In contrast to normoxic conditions, GE performed in hypoxia did not induce statistically significant changes in the concentration of any of the measured sphingolipids in either plasma or its fractions (Fig. 1). On the other hand, in erythrocytes the content of S1P and sphingomyelin decreased at the end of the exercise (Table 1). This effect, however, disappeared following 30 min of rest. A similar reduction in S1P level was observed in thrombocytes, but in contrast to RBCs, it persisted during the recovery period (Table 1). Hypoxic GE did not induce statistically significant changes in the rate of sphingoid base-1-phosphates release from erythrocytes (Fig. 3).

GE, either under normoxic or hypoxic conditions, did not induce statistically significant changes in the concentration of ceramide and dihydroceramide in plasma or its fractions (data not shown).

### Simulated 30 km individual time trial

**FIG. 2:**
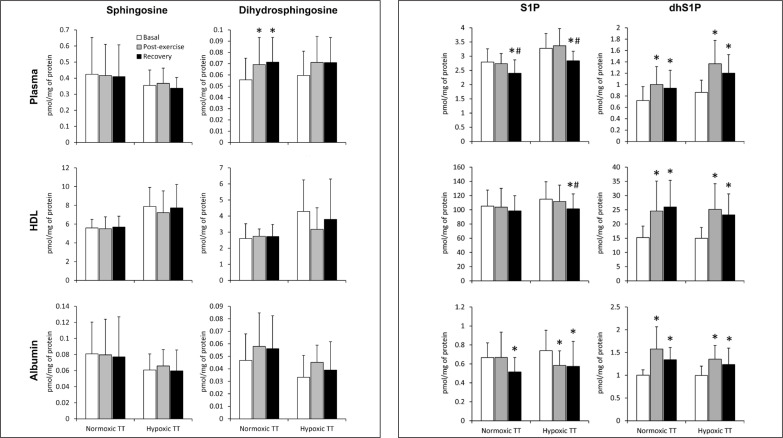
The content of sphingolipids in the plasma, as well as in its high-density lipoprotein (HDL) and albumin fractions sampled before, immediately after, and following 30 min of recovery from a simulated 30km individual cycling time trial (TT) performed either in normoxia or at a simulated altitude of 2,000m asl (n=15). The results are means ± SD. * – p<0.05 vs. the basal value, # – p<0.05 vs. the post-exercise value. HDL – high-density lipoprotein, dhS1P – dihydrosphingosine-1-phosphate, S1P – sphingosine-1-phosphate.

The TT performed under normoxic conditions induced an increase in the total plasma concentration of dihydrosphingosine and dhS1P that persisted during the recovery period. The same pattern of changes in the dhS1P level was observed also in the case of HDL- and albumin-bound fractions of this sphingolipid. On the other hand, the total S1P concentration in the plasma was reduced below the basal level following 30 min of recovery. This effect resulted from a decrease in the level of the albumin-bound S1P, since its content in the HDL-bound fraction did not show a significant change (Fig. 2).

**FIG. 3:**
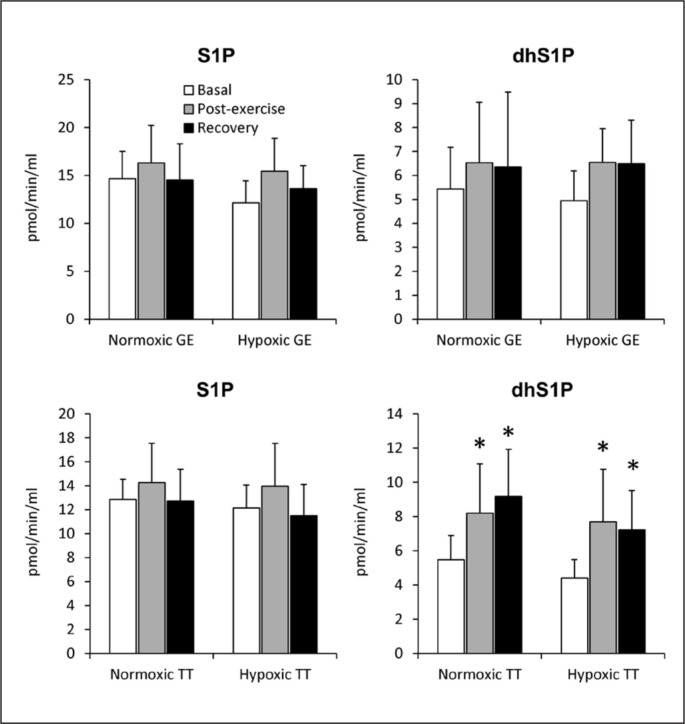
The rate of sphingosine-1-phosphate (S1P) and dihydrosphingosine-1-phosphate (dhS1P) release from erythrocytes sampled before, immediately after, and following 30 min of recovery from a graded cycling exercise until volitional exhaustion (GE) or a simulated 30km individual cycling time trial (TT) performed either in normoxia or at a simulated altitude of 2,000m asl (n=15). Notes: The results are means ± SD. * – p<0.05 vs. the basal value.

Similarly to plasma, the level of dihydrosphingosine and dhS1P in RBCs and thrombocytes increased in response to normoxic TT, and remained elevated during the recovery period (Table 2). The same pattern of changes was observed for the rate of dhS1P release from erythrocytes (Fig. 3). In addition, compared to the basal level, the erythrocyte S1P content was reduced, and platelet dihydroceramide was increased following 30 min of rest (Table 2).

**TABLE 2:** The content of sphingolipids in erythrocytes and platelets sampled before, immediately after, and following 30 min of recovery from a simulated 30 km individual cycling time trial (TT) performed either in normoxia or at a simulated altitude of 2,000 m asl (n = 15).

	Normoxic TT			Hypoxic TT		

	Basal	Post-ex.	Recovery	Basal	Post-ex.	Recovery
**Erythrocytes (pmol/mg Hb)**						
Sphingosine	0.498 ± 0.126	0.491 ± 0.084	0.553 ± 0.14	0.349 ± 0.068	0.353 ± 0.073	0.353 ± 0.066
dhSph	0.095 ± 0.037	0.134 ± 0.045^*^	0.151 ± 0.034^*^^#^	0.079 ± 0.027	0.127 ± 0.034^*^	0.121 ± 0.028^*^
S1P	6.53 ± 1.31	6.29 ± 1.1	5.93 ± 1.22^*^	5.26 ± 1.02	5.27 ± 1.15	4.91 ± 0.95
dhS1P	5.57 ± 0.89	8 ± 1.23^*^	7.91 ± 1.98^*^	3.66 ± 0.9	5.27 ± 1.38^*^	5.46 ± 1.36^*^
Ceramide	67.4 ± 15.5	69.6 ± 11.7	66.1 ± 13.9	52.1 ± 12.9	48.4 ± 11.5	49.9 ± 12.9
dhCer	9.73 ± 6.09	10 ± 5.23	9.51 ± 5.41	7.07 ± 4.43	5.98 ± 2.36	6.42 ± 3.9
Sphingomyelin	1267 ± 178	1141 ± 142	1174 ± 176	1349 ± 162	1311 ± 165	1295 ± 169

**Platelets (pmol/mg protein)**						
Sphingosine	51.7 ± 19.9	53.2 ± 20.6	60.6 ± 26.5	46.5 ± 15.2	40.8 ± 13.7	41.7 ± 18.1
dhSph	9.64 ± 4.66	13.2 ± 6.5^*^	15.28 ± 8^*^^#^	9.18 ± 3.88	9.74 ± 3.23	10.77 ± 4.32
S1P	651 ± 206	590 ± 153	640 ± 115	653 ± 170	584 ± 108	600 ± 148
dhS1P	231 ± 99	303 ± 98^*^	341 ± 87^*^	250 ± 79	300 ± 67^*^	334 ± 88^*^^#^
Ceramide	655 ± 204	600 ± 205	668 ± 305	672 ± 155	597 ± 160	614 ± 180
dhCer	366 ± 143	337 ± 143	404 ± 168^#^	407 ± 119	360 ± 89	351 ± 122
Sphingomyelin	21663 ± 5434	19328 ± 3566	21622 ± 3358	20251 ± 3319	18845 ± 2733	19371 ± 3827

The results are means ± SD.

* – p < 0.05 vs. the basal value,

# – p < 0.05 vs. the post-exercise value.

S1P – sphingosine-1-phosphate, dhCer – dihydroceramide, dhS1P – dihydrosphingosine-1-phosphate, dhSph – dihydrosphingosine, Hb – hemoglobin.

The TT performed in hypoxia induced an increase in the concentration of dhS1P in the plasma, as well as in HDL and albumin fractions. The level of this sphingolipid remained elevated during the recovery period. On the other hand, the total plasma concentration of S1P at this time point, as well as its content in both HDL and albumin fractions, was reduced below the basal value. In the case of the albumin-bound S1P, the reduction was observed also immediately after the exercise (Fig. 2).

Similarly to plasma, the level of dhS1P in erythrocytes and platelets increased in response to hypoxic TT, and remained elevated during the recovery period. In RBCs the same pattern of changes was observed also for the content of dihydrosphingosine (Table 2). The rate of dhS1P release from erythrocytes was increased by the TT performed in hypoxia, and remained elevated following 30 min of recovery (Fig. 3).

TT, either under normoxic or hypoxic conditions, did not induce statistically significant changes in the concentration of ceramide and dihydroceramide in plasma or its fractions (data not shown).

## DISCUSSION

In line with our previous results [8, 18], we observed that metabolism of circulating S1P and dhS1P was markedly affected by acute exercise in normoxia. However, in the present study these observations were, for the first time, extended by determination of sphingoid base-1-phosphates concentrations in their major plasma pools, and the rate of their release from RBCs. In addition, the effect of hypoxia on exercise-induced changes in sphingolipid metabolism has not been studied so far.

The total plasma S1P concentration was transiently increased following GE in normoxia, a similar response was observed in our previous report where athletes performed identical exercise protocol on a rowing ergometer [8]. Here we found that this effect results entirely from the increased concentration of the HDL-bound S1P, which is considered to be the plasma S1P pool with the highest biological activity [23, 24]. Interestingly, endurance training was also reported to selectively increase the HDL-bound S1P [9]. In line with our previous study [8], the GE-induced elevation in plasma S1P concentration was not associated with changes in its content, or the rate of its release, in blood cells, which argues against the involvement of either RBCs or platelets in this phenomenon.

On the other hand, the TT in both normoxia and hypoxia induced an increase in the total plasma dhS1P concentration, resulting from elevation in HDL- as well as albumin-bound pools. We found that this effect was a consequence of increased availability of dihydrosphingosine in the plasma, which caused accumulation of dhS1P in RBCs and platelets, and ultimately augmented dhS1P release to the circulation. In general, these results are in line with our previous study where athletes performed a similar submaximal exercise (60 min at 65% of VO_2max_) on a rowing ergometer [8]. However, in the present report the total plasma S1P concentration was reduced during the recovery period due to a decrease in the amount of the albumin-bound pool. A similar tendency was observed by Bergman et al. [25] in the serum of untrained subjects following 2 hours of recovery from a 90-minute exercise on a cycle ergometer. It should be noted that acute changes in plasma volume induced by exercise did not contribute to alterations in sphingolipid concentrations found in our study, since they were expressed on a protein, not volume basis.

We can only speculate on the origin of dihydrosphingosine released to the circulation during the TT. In our previous report, dihydrosphingosine was not found to be released across a leg either at rest or during exercise and recovery period [8]. However, our experiment on rats identified liver as a prominent source of plasma dihydrosphingosine [26]. It is, therefore, possible that the rate of its hepatic release increases during prolonged submaximal exercise.

As already mentioned in the introduction, Sun et al. [10] found that S1P levels in both plasma and RBCs are elevated following ascent to high altitude. However, in the present study, contrary to our initial hypothesis, hypoxia did not augment the exercise-induced increase in circulating sphingoid base-1-phosphate concentration. The response observed for dhS1P following TT was not affected by concomitant exposure to simulated altitude. Surprisingly, the increase in plasma S1P concentration induced by GE was abolished when the exercise was performed under hypoxic conditions. This effect was associated with a reduction in S1P content in erythrocytes and platelets, that was not observed in normoxia, which indicates that blood cells contributed to this phenomenon. In addition, hypoxia enhanced the reduction in plasma S1P concentration observed during the recovery from TT. First of all, the reduction was no longer limited to the albumin-bound pool, since a similar effect was found also for the HDL-bound S1P. Secondly, in hypoxia the decrease in the former pool developed already at the end of the exercise.

The lack of increase in the concentration of circulating S1P under exposure to simulated altitude could be explained by insufficient strength and duration of the hypoxic stimulus. In our study, the athletes spent up to 90 minutes at a simulated altitude of 2,000 m, whereas Sun et al. [10] exposed the subjects to 5,260 m for at least 12 h. Moreover, they showed that in mice exposure to a simulated altitude of 5,260 m lasting more than 6 h was required to increase the concentration of S1P in plasma and erythrocytes. The fact that in our study hypoxia tended to reduce the concentration of circulating S1P may seem puzzling. It should be noted, however, that D’Alessandro et al. [27] found that the time course of changes in erythrocyte S1P content during exposure to high altitude included a brief period of initial reduction preceding the subsequent increase.

Several studies showed that plasma S1P concentration decreases upon strong activation of inflammatory pathways. Such effect was observed following lipopolysaccharide administration to mice, as well as in patients with acute pancreatitis, sepsis, and COVID-19 infection [28–31]. In our recent paper including results obtained during the same experiment, we found that hypoxia markedly augmented oxidative stress and inflammatory response during both GE and TT [32]. It is, therefore, possible that these factors were responsible for the hypoxia-induced reduction in the post-exercise concentration of circulating S1P.

It is widely accepted that live high-train low (LHTL) model of altitude training is more effective in terms of athletic performance improvements than the live high-train high strategy [33]. In addition, an effective variation of the LHTL model has been developed, where athletes live and perform low-intensity training at moderate altitude and only high-intensity training takes place at low altitude [34]. Our observation that hypoxia abolishes the increase in plasma S1P concentration induced by high-intensity exercise supports the notion that in altitude programs this type of training should be performed at low altitude. Furthermore, considering the important role of extracellular S1P in muscle regeneration [7], our results raise a question as to whether hypoxic training should be implemented during recovery from injuries.

Sun et al. [10] found that elevation in the content of intracellular S1P plays a key role in hypoxia-induced increase in 2,3-bisphosphoglycerate (2,3-BPG) level in RBCs. The mechanism underlying this effect involves S1P-induced anchoring of deoxy-Hb to the plasma membrane resulting in enhanced release of membrane-bound glycolytic enzymes to the cytosol. We have recently reported that concentration of 2,3-BPG in RBCs sampled during the same experiment was reduced following the TT in hypoxia [35]. The effect developed despite the lack of changes in the content of S1P in erythrocytes. Our findings indicate that S1P is not involved in alterations in RBCs 2,3-BPG content induced by acute exercise in hypoxia.

S1P and dhS1P were shown to activate S1PRs with equal potency [36] and, therefore, it is commonly assumed that they induce similar effects. This is, however, not always the case. First of all, these two sphingoid base-1-phosphates are characterized by different distribution between plasma fractions and show distinct transport protein preferences [37]. Secondly, extracellular S1P was reported to have a much shorter half-life than dhS1P [38]. It was also found that dhS1P induces stronger S1PR-mediated reduction in intracellular cAMP concentration and higher magnitude of calcium mobilization compared to S1P [38, 39]. In some studies S1P and dhS1P were even shown to induce opposite effects [40, 41]. Interestingly, in our study plasma S1P concentration was increased only in response to a maximal exercise, whereas that of dhS1P only by prolonged submaximal effort. This observation suggests that S1P and dhS1P may be involved in different aspects of muscle adaptation to exercise. As already mentioned in the introduction, S1P emerged as an important regulator of muscle function [7]. However, the role of dhS1P in muscle biology has not been studied so far and represents an interesting area for further research.

## CONCLUSIONS

In summary, we found that simulated 30 km time trial increased dhS1P concentration in plasma (both HDL- and albumin-bound) and blood cells, as well as the rate of dhS1P release from erythrocytes, regardless of oxygen availability. The plasma concentration of S1P was, however, reduced during the recovery phase, and this trend was augmented by hypoxia. On the other hand, graded exercise until exhaustion in normoxia induced a selective increase of HDL-bound S1P in the plasma. This effect disappeared when the exercise was performed under hypoxic conditions, and it was associated with reduced S1P level in blood cells. We conclude that submaximal exercise elevates total plasma dhS1P concentration via increased availability of dihydrosphingosine resulting in enhanced dhS1P synthesis and release by blood cells. Maximal exercise, on the other hand, induces a selective increase in HDL-bound S1P, which is a consequence of mechanisms not related to blood cells. We also conclude that hypoxia does not affect the exercise-induced changes in metabolism of circulating dhS1P, but reduces post-exercise plasma S1P concentration. Therefore, extracellular S1P does not seem to be involved in adaptation to acute exercise in hypoxia.
